# Lectures on Visceral and Surgical Anatomy, and Operative Surgery

**Published:** 1843-07

**Authors:** 


### LECTURES ON VISCERAL AND SURGICAL ANATOMY, AND OPERATIVE SURGERY.

Professors Cobb and Gross will commence their fall course of lectures, on the above subjects, on the fifteenth of September, and continue them until the last Saturday in October. We would respectfully urge upon students of medicine the propriety of attending these lectures. They are upon topics, which are (whether right or wrong) almost wholly omitted in the usual winter course, and yet a knowledge of them is absolutely indispensable to a successful and honorable career as a practitioner. C.

